# Research supervisors’ views of barriers and enablers for research projects undertaken by medical students; a mixed methods evaluation of a post-graduate medical degree research project program

**DOI:** 10.1186/s12909-022-03429-0

**Published:** 2022-05-13

**Authors:** Joanne Hart, Jonathan Hakim, Rajneesh Kaur, Richmond Jeremy, Genevieve Coorey, Eszter Kalman, Rebekah Jenkin, David Bowen

**Affiliations:** 1 Sydney Medical School, Faculty of Medicine and Health, University of Sydney, Sydney, NSW Australia; 2 School of Health Sciences, Faculty of Medicine and Health, University of Sydney, Sydney, NSW Australia; 3 Office of the Deputy Vice Chancellor (Education), University of Sydney, Sydney, NSW Australia; 4 School of Medical Sciences, Faculty of Medicine and Health, University of Sydney, Sydney, NSW Australia

**Keywords:** Medical research projects, Student supervision, Research supervisors, Research supervision practice, Research skills development, Medical student projects, Student thesis, Scholarly research

## Abstract

**Background:**

Medical degree programs use scholarly activities to support development of basic research skills, critical evaluation of medical information and promotion of medical research. The University of Sydney Doctor of Medicine Program includes a compulsory research project. Medical student projects are supervised by academic staff and affiliates, including biomedical science researchers and clinician-academics. This study investigated research supervisors’ observations of the barriers to and enablers of successful medical student research projects.

**Methods:**

Research supervisors (*n* = 130) completed an anonymous, online survey after the completion of the research project. Survey questions targeted the research supervisors’ perceptions of barriers to successful completion of projects and sources of support for their supervision of the student project. Data were analysed by descriptive statistics and using manifest content analysis. Further quantitative investigation was made by cross-tabulation according to prior research supervision experience.

**Results:**

Research supervisors reported that students needed both generic skills (75%) and research-based skills (71%) to successfully complete the project. The major barrier to successful research projects was the lack of protected time for research activities (61%). The assessment schedule with compulsory progress milestones enabled project completion (75%), and improved scientific presentation (90%) and writing (93%) skills. Supervisors requested further support for their students for statistics (75%), scientific writing (51%), and funding for projects (52%). Prior research supervision experience influenced the responses. Compared to novice supervisors, highly experienced supervisors were significantly more likely to want students to be allocated dedicated time for the project (*P* < 0.01) and reported higher rates of access to expert assistance in scientific writing, preparing ethics applications and research methodology. Novice supervisors reported higher rates of unexpected project delays and data acquisition problems (*P* < 0.05). Co-supervision was favoured by experienced supervisors but rejected by novice supervisors.

**Conclusions:**

Both generic and research-related skills were important for medical student research project success. Overall, protected research time, financial and other academic support were identified as factors that would improve the research project program. Prior research supervision experience influences perceptions of program barriers and enablers. These findings will inform future support needs for projects and research supervisor training for the research supervision role.

## Introduction

Medical education programs increasingly employ scholarly activities to support development of basic research skills, the ability to critically evaluate medical information and the practice of evidence-based medicine [1]. Furthermore, research activities undertaken by students can foster life-long interest in medical research [2–4]. This is crucial for the development of clinician-academics, who have key roles in clinical research and translational medicine [5]. There are declining numbers of clinician-academics in Australia [6] and globally [7], thus the importance of fostering interest in research in medical students is imperative.

The University of Sydney 4-year post-graduate Doctor of Medicine Degree (MD) is unique, enrolling students from a wide range of previous academic backgrounds and with various prior research and employment experiences. As an integral and compulsory component of the MD Program curriculum, the research project (MD Project) is delivered as 320 study hours over 2.5 years, from mid-Year 1 to the end of Year 3. Students receive 40 h of training in research methods, basic statistics and research ethics at the end of Year 1, shortly after they commence their projects. Students complete their research project on top of the overall MD program curriculum, without protected research time.

The pedagogical framework for the MD Project program employs active, experiential, project-based learning in a research context with individual projects being supervised by academic staff mentors or mentoring teams. The intended learning objectives of the MD Project are summarised in Table 1.

**Table 1:** MD Project Learning Objectives

MD Project Learning Objectives
1. Formulate a research question, hypothesis, or issue for investigation
2. Identify, obtain and integrate existing knowledge relevant to the research question or hypothesis
3. Organise and conduct a research project
4. Collect and analyse data and logically present the findings
5. Prepare a scientific report that draws appropriate conclusions from the findings, recognises the strengths and limitations of the design and methods of the project and considers the findings in light of current knowledge in the area

In the course of the research project, students need to develop key research and generic skills, including self-motivation, time management and organisation, and building relationships in clinical and research laboratory environments. Students achieve these aims through hands-on experience in devising and conducting a project relevant to health or medicine, analysing the findings, and reporting the results. The scope of MD Projects is broad and includes clinical studies, projects in biomedical science, epidemiology and public health, medical education, bioinformatic and information technology and policy, law, and ethics. A series of compulsory milestone assessments are designed to facilitate progress of each project towards completion. These Milestone tasks include an early project outline, a full appraisal of the ethical implications of the project and verification that ethics approval has been obtained, a structured literature search strategy, and progress reports involving written and oral scientific presentations. The final assessment task is a 3000-word scientific report. Many students were encouraged to present at conferences or prepare manuscripts for peer-reviewed journals, however these were not requirements of the MD Project program. Students are supervised individually or in small groups (usually 2–5 students) by academic staff and affiliates, including basic research scientists, public health researchers and clinicians. The majority of supervisors are not directly employed Faculty members, but University affiliates, who are not specifically remunerated for their time. Supervisors were not required to have a PhD or any formal research supervision training. No Faculty funding was provided to support the project or its supervision. Supervisors were required to provide all project materials and expertise and would supervise up to 6 students at a time. Many supervisors were based in public hospitals and took on MD Project supervision in addition to their existing clinical and/or research workloads.

The research supervisor has a key role in the success of this traditional model of research project [8]; however, research supervision experience varies from very limited to extensive. Although research supervision training for supervisors of higher degree students is common worldwide and often mandatory, most academics learn to supervise research students “on-the-job” and by emulating their own research mentors [9]. Currently, there is no formal training provided for the supervisors of MD Projects, or for those supervising similar short-term research projects by undergraduates, including Honours degrees [10]. Whilst there is evaluation data available for similar research project programs from the students’ viewpoint [11–14], the perspective of the supervisors is under-reported. Given the key role of the supervisor in this research education model, their experiences are an important source of information to guide future program improvements.

This study sought the views of research supervisors on the MD Project program and their experiences of supervising medical student research projects, including:

- observations on the barriers to and enablers of successfully completing an MD Project,
- sources of support for their supervision of the project,
- extent of research supervision experience on attitudes to and overall experience of supervising the MD projects,
- requirements for professional development or other assistance.

## Materials and methods

### Study Design

This study is a mixed methods evaluation of the MD Project program from the perspective of the research supervisors.

### Participants

MD Project research supervisors were invited by email to complete an anonymous online survey following the completion of the student projects in 2018 and 2020. Participants were University academic staff and appropriately qualified affiliates, including basic research scientists and clinicians. Participants had varying levels of previous research supervision experience, ranging from none to supervision of post-graduate research degree completions. Their areas of research expertise were broad though based in health and medical research. There were no exclusion criteria. Consent to participate was inferred if participants opted to complete and submit an online survey.

### Survey tool

The survey tool was developed specifically for this program and was reviewed and refined by MD Program Faculty members, including some research supervisors, to optimise face and content validity. The survey consisted of 30 items, mostly on a 5-point Likert-type scale (with responses of *not at all, slightly, moderately, very, extremely*), with optional text responses. Some items required selection of multiple responses from a given list. Survey domains included participant demographics and prior research supervision experience, their overall experience of supervising MD Projects, enablers for and barriers to successful completion of the project (at the MD Program, project, supervisor and student level) and resources and support needed for the supervision role. Participants were provided with a link to the survey within an email invitation to participate; responses were anonymous and aggregate data are shown. The survey tool is available upon request from the authors.

### Data analysis

Descriptive analyses were used to explore the overall patterns of response, and qualitative content analysis was used to examine the responses to open ended questions. MD Project supervisors were divided into three categories of research supervision experience: novice (no prior research supervision), moderately experienced (supervised any one of: summer research projects of duration 6–10 weeks, undergraduate Honours projects of up to 6 months or post-graduate research degrees) and highly experienced, (all of the abovementioned supervision types) based on their responses to the survey. Descriptive analysis and Chi Square test including the Mantel–Haenszel test of trend were used to assess any differences in responses between the supervisor experience groups. *P* < 0.05 was accepted as statistically significant. Quantitative analyses were carried out in SPSS V26 (IBM Corp, Armonk, NY). Responses from open ended questions were analysed through qualitative methods using manifest content analysis [15]. Initially deductive coding for explicit phrases was carried out. These codes were then contextualised with the research question of the study. This was followed by generation of homogenous categories from the codes. Conclusions were drawn through investigator triangulation.

## Results

### Response rates and Faculty respondent demographics

Survey responses were collected from two cohorts of MD Project supervisors, following the completion of the project. From 463 MD Project supervisors’ invitations, 130 (28%) responded. Most respondents identified as clinical researchers, followed by public health academics and biomedical science researchers (Table 2). Others had expertise in medical education, bioinformatics, information technology and health-related policy, law, or medical ethics. Many identified multiple areas of expertise.

**Table 2:** Faculty areas of research expertise and prior research supervision experience

Specialty Area^a^			n	%
**Clinical Research**			86	66
**Public Health and Epidemiology**			43	32
**Biomedical Science**			38	29
**Medical Education**			22	16
**Bioinformatics and Information Technology**			11	9
**Medical Policy, Law or Ethics**			11	9
**Supervisor experience**	**Supervision experience type**	**Project duration**	**n**	**%**
**Novice**	None	-	13	10
**Moderately experienced**^**a**^	*Summer research projects*	*6–10 weeks*	*5*	*4*
	*Honours projects*	*6 months*	*12*	*9*
	*Post-graduate completions*	*2–4 years*	*22*	*17*
	Moderately Experienced Total		45	34
**Highly experienced**	All the above supervision types	-	75	56

^a^Multiple responses allowed

### Faculty research supervision expertise

MD Project supervisors are required to be academic staff or affiliates of the University, however there are no other specific requirements to become a supervisor. Approximately 60% of the respondents were highly experienced research supervisors across a range of project types and duration. One third had moderate experience supervising either summer research project students, Honours Degree students or post-graduate research students. Ten percent of the supervisors had no previous research supervision experience (Table 2).

### Student-Supervisor relationship

**Fig. 1:**
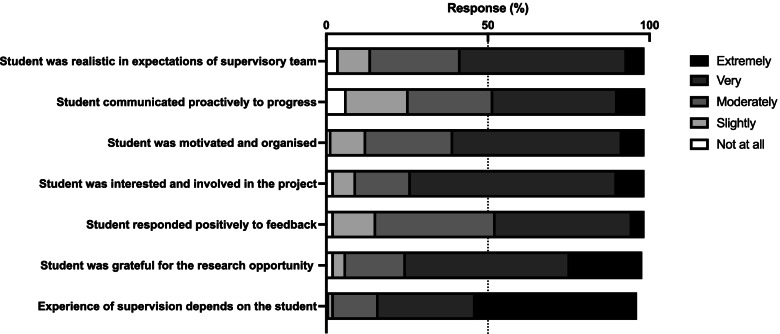
Student-Supervisor relationship items. Supervisors responded to a number of items related to the student-supervisor relationship on a Likert scale from not at all to extremely. Percentage of responses are shown

About half (47%) of the supervisors felt their feedback on student performance was only moderately well received (Fig. 1), though the majority (73%) of supervisors felt the students were grateful for the opportunity to do research with their team (Fig. 1). Supervisors reported that students were organised and interested in their projects and were moderately proactive in communications. Overall, there was agreement amongst MD Project supervisors (86%) that their experience of supervision was very dependent on the individual student (Fig. 1).

### Enablers for successful completion of the research project

#### Student skills needed to successfully complete the MD project

**Fig. 2:**
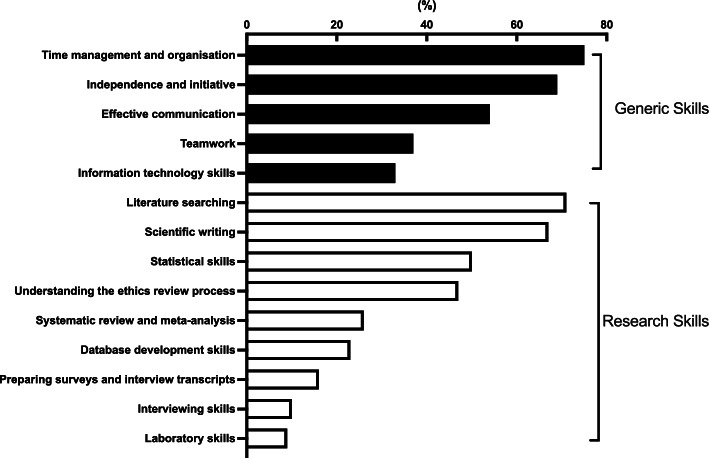
Research supervisors’ perceptions of skills students needed for completing research projects. Percentage of supervisors (*n* = 130) who selected these items from a list of generic and research skills needed in the student research project

Respondents identified skills that students needed for successfully completing their MD Project, these are presented in Fig. 2. These were often generic skills, including time management and organisation, independence and initiative and effective communication skills. The top research skills needed included literature searching, scientific writing, statistical skills and navigating the ethics review process (Fig. 2). Task-specific skills such as familiarity with information technology and databases were considered less critical, which may reflect the mix of projects undertaken.

#### Assessment schedule

There were mixed views on the utility of the milestone assessment tasks, which are presented in Table 3. The majority of respondents reported that compulsory milestone assessment tasks helped students make progress on their project, though only half thought the tasks were necessary to maintain momentum or hold students accountable to the standards required. About one-third reported that milestones assured the supervisor that the student was progressing as expected. Novice supervisors generally rated the assessment tasks as more useful than the experienced supervisors (Table 3). The oral presentations were rated as very useful for student progress, helping them learn to accept and respond to feedback and develop their scientific presentation skills. Preparing a final scientific report was strongly viewed as a very useful activity (Table 3).

**Table 3:** Assessment tasks and whether they facilitated MD Project progress

Assessment Tasks	All supervisors^a^ % Agree (n)	Novice supervisors^b^ % Agree (n)
**Milestone Assessment Tasks**		
Helped project progress	75 (96)	85 (11)
Necessary to maintain project momentum	56 (72)	69 (9)
Held students accountable to the standard required	49 (62)	62 (8)
Assured the supervisor of adequate student progress	37 (48)	39 (5)
**Oral presentations**		
Important for student progress	86 (107)	100 (12)
Helped students learn to accept and respond to feedback	84 (104)	100 (12)
Important for learning scientific presentation skills	90 (111)	92 (11)
**Final Scientific Report**		
Writing a scientific report was a valuable skill	93 (116)	92 (11)

^a^Not all participants answered all questions

^b^Novice supervisors had no research supervision experience prior to the MD Project

### Barriers to successful completion of the research project

**Fig. 3:**
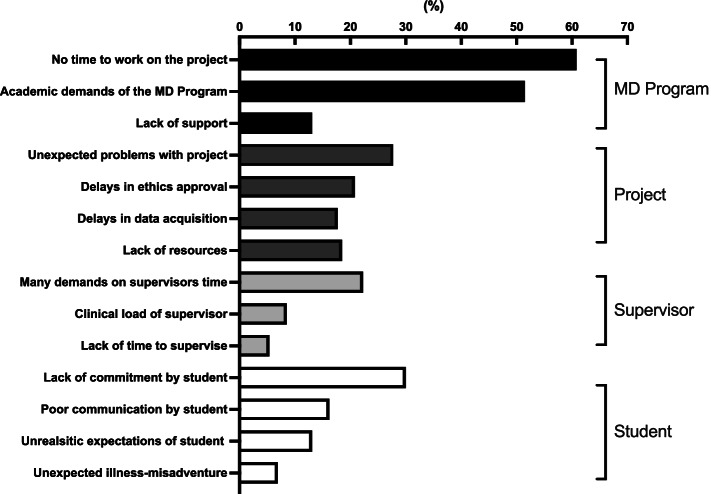
Barriers to successful completion of MD Projects reported by supervisors. Percentage of supervisors (*n* = 130) that selected these items from a list of barriers to successfully completing the research project. These barriers were grouped in relation to the MD Program, the project, the supervisor or the student

Potential impediments for MD project success fell into four broad groups: Program level, project level, supervisor-related and student-related (Fig. 3). The principal barriers were at program level, with lack of dedicated time for the project and competing academic demands on students of the overall MD Program being most frequently cited (Fig. 3). At project level, unexpected problems, such as delays in data acquisition and time taken for Ethics Committee review and approval were reported. Supervisor time constraints reflected clinical load and other demands. Lack of previous research experience, or lack of commitment to the project were student-related characteristics that were identified as important barriers.

Challenges described by MD Project supervisors in free text responses indicated a range of other concerns mostly related to student issues but also to their own role as supervisor. They report that a major challenge for the students was competing priorities for learning. The MD Project Milestone tasks therefore became extrinsic motivators and barriers to overcome instead of activities that meaningfully contributed to their learning. This was particularly evident in students competing milestones ‘just in time’ leaving little opportunity for meaningful feedback from supervisors. Other difficulties cited were students having no research or science background as reflected in the following quotes:

*“The students struggle to maintain any momentum with their MD Projects as they prioritise other aspects of the MD Program and other deadlines (naturally), so the MD Project often is done all in a rush near the milestone deadlines which is then challenging for supervisors to find the time for a large number of students who need help.” (Experienced Supervisor, Epidemiologist)*

*“Most (students) have a poor understanding of research and stats. This was especially the case with one student from a non-science background.” (Moderately Experienced Supervisor, Clinician)*

Challenges cited for MD Project supervisors included the demands of completing other parts of the course and MD Project simultaneously, demanding or disengaged students, a large number of students to supervise, and a lack of time or competing priorities or deadlines. It was reported by some that this type of project supervision was not a good fit for a full-time researcher.

*“Of the 11 students I have been involved with, even though all have done well many are very disengaged until the last week or two of the projects, then very demanding for input into their report.” (Experienced Supervisor, Clinician & Biomedical Researcher)*

*“The students have so many competing demands that the MD Project is a real challenge for everyone. As a full-time researcher, fitting such students into my main program is not a good fit.” (Experienced Supervisor, Clinician)*

### Supervision support for MD projects

**Fig. 4:**
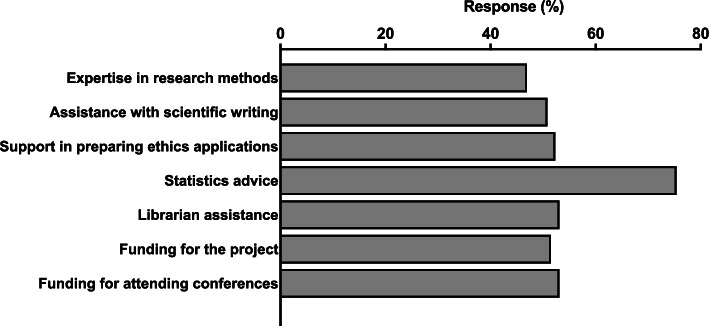
Support and resources needed by MD Project supervisors. Percentage of supervisors (*n* = 130) that selected these items from a list of supports and resources needed for the MD Project

Only 11% of respondents said they had all the resources they needed to run the MD Project. The respondents indicated that more support was required for statistics, ethics applications, scientific writing, research methods, and funding both for the project costs and for students to attend conferences (Fig. 4).

### Effect of prior research supervision experience on responses

Prior research supervision experience did not affect the perception of the generic skills that supervisors felt students needed to successfully complete their MD Project. However, skills that were more highly regarded by novice supervisors included skills in literature searching (92%), database development (46%) and understanding the ethics review process (69%). Highly experienced supervisors were more likely to cite independence and initiative (75%) as a required skill than novice supervisors (47%). It is notable that novice supervisors recorded a higher agreement with the utility of the assessment tasks than the overall respondent data (Table 3). Regarding the student-supervisor relationship, there was no difference in responses by prior research supervision experience.

**Fig. 5:**
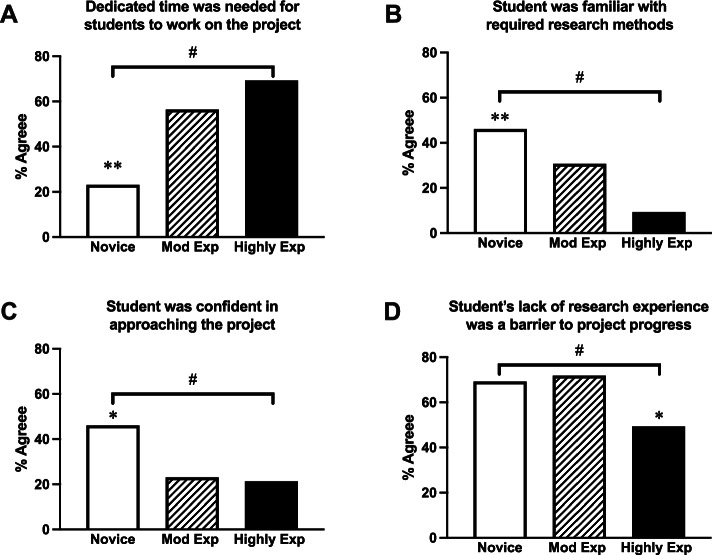
Novice supervisors’ appraisal of student research capabilities. **A** Novice supervisors were significantly less likely to want a dedicated time for the project, (**B**) were more likely to consider their students familiar with research methodology and (**C**) confident in approaching the project. **D** Highly experienced supervisors were significantly less likely to cite their student’s lack of previous research experience as a barrier compared to moderately experienced and novice supervisors. * *P* < 0.05, **P < 0.01, χ^2^-test; # = *P* < 0.05, Mantel–Haenszel test of trend, by supervisor experience

Interestingly, although overall the major barrier cited was a lack of dedicated time for the MD Project (Fig. 3), novice supervisors were significantly less likely to want a dedicated time for the project (23%) compared with highly experienced supervisors (69%, χ^2^ = 10.351, *P* = 0.005 Fig. 5A). Lack of dedicated time for the MD Project was recognised as a barrier which increased with supervision experience (Mantel–Haenszel test of trend, *P* = 0.002, Fig. 5A). Further, highly experienced supervisors were significantly less likely to identify the student’s lack of previous research experience as a barrier (49%) compared to moderately experienced (72%) and novice supervisors and this trend was statistically significant (69.2%, χ^2^ = 6.040, *P* = 0.049). A significant trend of this being less of a barrier was noted with increasing supervision experience (Mantel–Haenszel test of trend, *P* = 0.031, Fig. 5D). Novice supervisors were significantly more likely to rate their students at the outset of the project as being familiar with research methods (χ^2^ = 13.431, *P* = 0.001). A significant trend was noted for this rating by supervision experience (Mantel–Haenszel test of trend, *P* = 0.005, Fig. 5B). Novice supervisors also felt that students were more confident in approaching their project than experienced supervisors and this trend was statistically significant (χ^2^ = 6.348, *P* = 0.042) and associated with supervision experience (Mantel–Haenszel test of trend, *P* = 0.046, Fig. 5C). No novice supervisors reported they had a lack of time for supervision, although novice supervisors identified their clinical load as a barrier (15%) more often than experienced supervisors (8%).

**Fig. 6:**
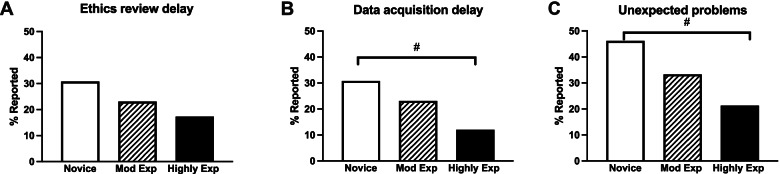
Novice supervisors’ reported rates of project delays or problems. MD Project delays, due to (**A**) ethics approval, (**B**) data acquisition or (**C**) unexpected problems were more often reported with novice supervisors, with a decreasing trend in delays as supervision experience increased (# = *P* < 0.05, Mantel–Haenszel test of trend, by supervisor experience)

Notably, compared to experienced supervisors, novice supervisors reported higher rates of project delays due to ethics committee review (χ^2^ = 1.463, *P* = 0.481, Fig. 6A) where a trend by supervision experience is observed but does not reach statistical significance. They also report increased rate of data acquisition problems (χ^2^ = 4.026, *P* = 0.134, Fig. 6B), and unexpected project problems (χ^2^ = 4.359, *P* = 0.113, Fig. 6C). A significant trend was observed by supervision experience (Mantel–Haenszel test of trend, *P* = 0.047, Fig. 6B, *P* = 0.038, Fig. 6C). Highly experienced supervisors reported significantly higher rates of access to expert assistance particularly in scientific writing (novice 7.7% vs highly experienced 21.3%, χ^2^ = 8.251, *P* = 0.016), and there was a significant trend with supervision experience (Mantel–Haenszel test of trend, *P* = 0.005). In addition, highly experienced supervisors reported twice the access to expertise for preparing ethics approval applications (novice 15.4% vs highly experienced 37.3%) and research methodology advice (novice 15.4% vs highly experienced 38.7%) compared to novice supervisors, though this does not reach statistical significance. Those with moderate prior supervision experience were significantly more likely to want orientation sessions for the MD Project (χ^2^ = 8.519, *P* = 0.014). None of the novice supervisors wanted co-supervision and few sought increased involvement of expert advisors (8%), whereas moderately and highly experienced supervisors were open to these options (16–20%).

Content analysis of free text comments revealed differences in perceptions of the contributions of supervisors to the MD Project program. The more experienced supervisors felt they had a responsibility to participate in the MD project as supervisors, with specific reference to the need for experience to support the student cohort and the difficulty of the task. Novice supervisors noted that they were gaining professional skills as a result of supervising the students. Thus, experienced supervisors felt they were giving something to the program, whereas novice supervisors felt they themselves received a benefit from the program.

*“For us who are experienced supervisors, we need to do this to help out the Faculty and the MD program. This is not for inexperienced supervisors.” (Experienced Supervisor, Clinician)*

*“There are a large number of students and relatively few tutors with research experience, so I feel there is a responsibility to participate.” (Experienced Supervisor, Biomedical Scientist)*

“Rewarding yet challenging at the same time. Helps with ongoing education and professional development for myself.” *(Novice Supervisor, Clinician)*

## Discussion

This study examines a large post-graduate medical student research project program from the perspective of the research project supervisors. Supervisors reported that students needed both generic skills and research-based skills to successfully complete the project. Across 3 years of the program, the students are expected to spend 320 h dedicated to their research project. Supervisors reported that having no protected time for research activities was a significant barrier to the successful completion of the project. Further support was requested for statistics, scientific writing and funding for projects. Importantly, prior research supervision experience affected the responses, where novice supervisors reported higher rates of project delays due to ethics review, data acquisition problems and unexpected project problems compared to experienced supervisors. Inexperienced supervisors also reported less access to supports, suggesting further support and training of novice supervisors would be of benefit.

The supervisor workforce in this study was mostly clinician researchers, followed by public health and epidemiology researchers and biomedical scientists. A smaller proportion of the supervisors oversaw medical education, bioinformatics, information technology or medical policy law or ethics projects. Thus, the project scope and supervisor research expertise varied, and many indicated they had multiple areas of expertise. This is in line with most medical degree scholarly programs which offer a wide scope of project experiences [2, 16–18]. Most of the respondents identified as being experienced supervisors, a third had supervised some project models, and some had no prior research supervision experience. This is common across student research programs, where the role of project supervisor often requires no qualification other than being a researcher or being available, though it is known that the supervision role requires support [16]. This study also provided some insight into the motivations of the research supervisors, where the experienced supervisors felt the need to contribute to teaching, whereas the novice supervisors wanted to gain supervision skills.

An important finding of this study is that supervisors report that both generic and research skills are important for successful completion of MD Projects. Indeed many of the generic skills needed are also required by medical professionals, and such skills are now routinely included in many medical program curricula [19]. These skills include time management and organisation skills, taking initiative and acting independently, and effective communication skills which all contribute to the development of professionalism [20].

The major barriers to student success identified by supervisors are similar to those previously published [21] comprising the trio of time, funding and the student-supervisor relationship. The delivery of the MD Project, within the already busy medical school curriculum, was cited as one of the major barriers for student success in their projects. A recent realist review also concluded that research experience for medical students required protected time and adequate supervision to achieve scholarly outcomes [4]. Interestingly, MD Project supervisors reported that students had time to complete their projects, although a lack of dedicated time to conduct the project manifested in students adopting a stop-start approach to their projects, as they navigated the rest of the medical program content. This was very clear in the respondent reports regarding student communication, which describe many students as being proactive only as milestone assessment tasks approached. The progressive assessment schedule for the MD Project was well received by the supervisors, who found it useful to progress projects, though only half thought milestone assessments were useful to maintain momentum of the projects or to determine how their students were tracking within the cohort (Table 3). Traditional scientific research project assessments were used, including written and oral progress reports and a final written scientific report, which were all considered very useful in project progress towards completion.

Only 11% of the supervisors said they had all the resources they needed to run their project; this is a clear area for improvement. The supervisory role was not remunerated, there was no backfill for time taken, no project funds available and nearly all supervisors had busy and demanding research and/or clinical roles. Thus, the volunteer nature of the supervisor cohort is quite important, especially given that some of the usual paybacks of supervising students to do research are uncommon in this setting, e.g., generating publications, piloting projects or advancing parts of larger projects. Supervisors reported that academic support for students in statistics, research methods, scientific writing and ethics were lacking and that central support for these services would be welcome. Thus, to sustainably run a research program like this at scale, further central support for these activities needs to be provided.

Participants were from a variety of specialty areas, both clinical and non-clinical, and with varying degrees of research supervision experience. Notably some survey responses were significantly different according to the respondent’s previous supervision experience. This is in line with a recent report [22] and trends with prior supervision experience were further explored. Novice supervisors were significantly more likely to rate their students at the outset as being familiar with research methods and confident in approaching their project. This likely reflects the supervisor’s inexperience and is consistent with previous reports that interpreting student understanding is difficult for novices [23]. They also may have different pre-existing expectations of the research project process than the experienced supervisors [22]. Novice supervisors were significantly less likely to report that a dedicated time was needed for students to work on the project, and this is contrary to consistent evidence that protected research time is required for the success of these projects [20]. A further finding is that highly experienced supervisors were significantly less likely to suggest that student’s lack of prior research experience was a barrier to project progress, possibly as they had better support structures in place for their students, and better understanding of how to guide students in their research activities.

Further, novice supervisors were more likely to report significant project delays, due to unexpected problems, ethics review, and data acquisition delays. In addition, there was a significant trend in these delays with prior supervision experience, suggesting that mentoring or further support for new supervisors would be useful to bridge the gap. Moreover, there was a significant trend showing that students of novice supervisors had less access to support for scientific writing, expertise in research methods and preparation of ethics review applications, further revealing areas where increased training and support would be useful for novice supervisors.

Quality research supervision involves expertise of the supervisor in the research area, and a willingness to guide the student through the research project process [24]. Different models of supervision are likely to be required for different students and different project types [25]. Further, studies show that the student-supervisor relationship is largely dependent on how reliant the student is on their supervisor; thus, students who are more dependent may need a different approach to supervision than those who are independent [26]. This is consistent with the current findings that supervisors felt that the overall supervision experience varied widely. The ideal research environment for medical students has been reported to involve individual supervision with continuous feedback [8]. Notably, many MD Project supervisors felt that their feedback on student performance was only moderately well received, but the reasons for this are not clear. Compiling and delivering feedback to assist student progress is a complex process with several considerations including the emotional impact of receiving or giving written feedback; written feedback in the supervisory power dynamic; communicating written feedback; and the content and structure of written feedback [27]. These proficiencies are a further area for future training considerations. In addition to this, improving the supervisor experience would likely cultivate future supervision capacity and retention of experienced supervisors, which is an important consideration for the sustainability of a large MD Project Program.

Many research supervisors are not specifically trained in the pedagogy associated with supervision. Although specific training programs have become standard for higher degree supervisors [9, 28], this is not the case for research supervision at the undergraduate or post-graduate coursework level, as in this program. Higher degree supervisor training programs cover topics like managing the relationship between student and supervisor, keeping roles and expectations clear, managing milestones and project progress. Other important considerations may be handling breakdowns in relationships, authorship, and research ethics issues [9, 29]. All of these are relevant to the MD project supervision. In this study, supervision experience ranged from none to extensive, but supervisors were not required to have any supervision qualifications. Notably, inexperienced supervisors were less inclined to have a second supervisor or expert content advisor involved in supervising their student’s project, whereas experienced supervisors were more open to this option. This finding is in accord with the supervisor professional identity dilemma previously reported for both novice and more experienced supervisors [23].

### Limitations

This cross-sectional study has limitations in that it is subject to self-report bias and the timing of the survey which took place at the end of the 2.5-year project risking the introduction of recall bias. The relatively low response rate (28%) reflects the participant cohort which includes busy clinicians and researchers [30].

## Conclusions

In conclusion, research supervisors reported that both generic and research-related skills were important for research project success. Overall, supervisors considered that the program delivered on its objectives, and that the assessment tasks enabled project progress and skill acquisition. Protected research time, funding, and academic support, particularly for research methods and ethics, would improve the research project program. Supervisor perceptions differed depending on prior research supervision experience and suggest a targeted training program could be beneficial. This should be further investigated to inform future support provisions.

## Data Availability

The datasets generated and/or analysed during the current study are not publicly available, as per conditions of Ethics Committee approval, but are available from the corresponding author on reasonable request.
